# Prognosis of Regenerative Endodontic Procedures in Mature Teeth: A Systematic Review and Meta-Analysis of Clinical and Radiographic Parameters

**DOI:** 10.3390/ma14164418

**Published:** 2021-08-06

**Authors:** Pantaleo Scelza, Fabiano Gonçalves, Isleine Caldas, Fernanda Nunes, Emanuelle Stellet Lourenço, Sandro Tavares, Marcela Magno, Andrea Pintor, Pietro Montemezzi, Emanuele Di Edoardo, Carlos Fernando de Almeida Barros Mourão, Gutemberg Alves, Miriam Zaccaro Scelza

**Affiliations:** 1 Geriatric Dentistry Department, Universidade Federal Fluminense, Niterói 24020-140, RJ, Brazil; pantaleoscelza@terra.com.br (P.S.); isleinecaldas@hotmail.com (I.C.); nandasouzanunes@gmail.com (F.N.); 2 Post-Graduate Program in Dentistry, Universidade Federal Fluminense, Niterói 24020-140, RJ, Brazil; fabianopalmeira@hotmail.com (F.G.); emanuelle_stellet@yahoo.com.br (E.S.L.); sandro.tavares04@gmail.com (S.T.); 3 Department of Pediatric Dentistry and Orthodontics, Universidade Federal do Rio de Janeiro, Rio de Janeiro 21941-902, RJ, Brazil; marcela.magno@hotmail.com (M.M.); andrea_pintor@hotmail.com (A.P.); 4 Independent Researcher, 24128 Bergamo, Italy; m.montemezzi@libero.it; 5 Independent Researcher, 20100 Milan, Italy; emanueledr.diedoardo@libero.it; 6 Clinical Research Unit of the Antonio Pedro Hospital, Universidade Federal Fluminense, Niterói 24033-900, RJ, Brazil; gutopepe@yahoo.com.br; 7 Laboratory of Experimental Culture Cell (LECCel), Department of Endodontics, Faculty of Dentistry, Universidade Federal Fluminense, Niterói 24020-140, RJ, Brazil

**Keywords:** dentition, permanent, meta-analysis, regenerative endodontics, systematic review

## Abstract

This work aimed to investigate the use of Regenerative Endodontic Procedures (REP) on the treatment of pulp necrosis in mature teeth through systematic review and meta-analysis of evidence on clinical and radiographic parameters before and after REP. A search was performed in different databases on 9 September 2020, including seven clinical studies and randomized controlled trials (RCT). The methodological quality was assessed using Revised Cochrane risk-of-bias (RoB 2) and Before-and-After tools. Meta-analyses were performed to evaluate the success incidences regarding the reduction of periapical lesion and recovery of sensitivity. The certainty of the evidence was assessed using GRADE. Meta-analysis showed a high overall success of 0.95 (0.92, 0.98) I^2^ = 6%, with high periapical lesion reduction at 12 months (0.93 (0.86, 0.96) I^2^ = 37%) and by the end of follow-up (0.91 (0.83, 0.96) I^2^ = 13%). Lower incidences of positive sensitivity response were identified for the electrical (0.58 (0.46, 0.70) I^2^ = 51%) and cold tests (0.70 (0.54, 0.84) I^2^ = 68%). The calculated levels of REP success were similar to those reported for immature teeth. With a very low certainty of evidence, the meta-analysis showed a high incidence of REP’s success for mature teeth with necrotic pulp evidenced by periapical lesion reduction and moderate positive responses to sensitivity tests.

## 1. Introduction

Pulp necrosis is caused by the death of pulp connective tissue, including the deterioration of blood and lymphatic vascularization and existing nerve fibers [1], affecting both immature and mature teeth. Conventional endodontics already have well-established treatment protocols using intracanal medication and filling materials in the root space [2,3]. However, it should not be ignored that teeth without vital pulp lose their defensive capacity, and when submitted to conventional endodontic treatment, may become increasingly vulnerable to external forces and susceptible to fractures and reinfections [4,5].

Ostby (1961) carried out studies involving biological agents such as blood clots, leading to what is now known as Regenerative Endodontics [6]. The American Association of Endodontists (AAE) [7] defines Regenerative Endodontics as “biologically based procedures designed to replace damaged structures, including dentin and root structures, as well as cells of the pulp–dentin complex” [8].

Regenerative Endodontics employs the tissue engineering triad (stem cells, biomimetic scaffolds, and bioactive growth factors) in the root canal space to regenerate the pulp tissue [9,10]. Regarding the cellular component of this triad, dental-derived stem cells can differentiate into odontoblast-like cells and act to maintain pulp homeostasis. These cells have high plasticity and pluripotency and are mainly available in biological niches associated with capillaries and nerve networks in the central, cell-rich zone region of the pulp [11]. Their secretome is comprised of several molecules, including trophic factors as chemokines, cytokines, growth factors, and hormones, sometimes included in extracellular vesicles [12], which have been widely considered as the main responsible for the regenerative properties of dental-derived stem cells related to their potential use on regenerative medicine [13]. Secreted mediators such as Bone Morphogenetic Protein 2 (BMP-2), Transforming Growth Factor Beta (TFG-β), and others may even become entrapped on the mineralized extracellular matrix (dentin), and available to act as regenerating factors upon induced demineralization during endodontic procedures [8]. Conversely, this microenvironment also influences the self-renewal and differentiation of dental-derived stem cells through a combination of biochemical, biophysical, and biomechanical factors.

In bioengineering, a favorable microenvironment of interaction between stem cells and a biocompatible scaffold is a crucial factor for tissue neoformation. Therefore, it is particularly important to establish a specific biological niche capable of offering adhesive, attractant, and proliferative conditions for stem cells, guiding and facilitating tissue repair [13,14]. Recent studies propose the use of scaffolds from the most varied origins, from alloplastic combinations of a polymer matrix, such as polylactic acid, with one or more bioactive or biointeractive components [14], to the use of autologous materials as biological scaffolds [15]. In this sense, the blood clot may act both as a natural scaffold and a provider of stem cells and growth factors from the apical papilla [15]. As a scaffold, it supports the retention, proliferation, migration, and organization of the spatial population of cells required for structural and functional term replacement of the target tissue [16]. Other autologous blood derivatives such as platelet-rich plasma (PRP) and fibrin-rich plasma (PRF) have been used within the root canal, inducing tissue regeneration [17]. Platelet concentrates contain key growth factors that stimulate collagen production, recruit other cells to the site of injury, induce anti-inflammatory agents, initiate vascular ingrowth, induce cell differentiation, and improve the soft and hard tissue wound healing potential into canal space. Therefore, they may become advantageous in cases of insufficient bleeding from periapical tissues [18]. Notwithstanding the distinct methodologies, blood clots and PRF showed similar effects regarding outcomes such as reducing periapical lesion, inducing apical closure, and increasing root length and thickness of immature teeth [19]. The protocol proposed by AAE in 2018 includes autologous materials such as blood clots, PRF, PRP, and autologous fibrin matrix (AFM) for teeth with necrotic pulp and immature apex [20].

Consistent data regarding the use of Regenerative Endodontic Procedures (REP) in immature teeth with necrotic pulp have been produced [19,21], and the emerging evidence may provide indications for a more assertive position regarding revitalization procedures [22]. Nevertheless, this discussion remains relatively limited in the scientific literature regarding the application of REP to mature teeth with necrotic pulp [23]. Anatomical differences from adult teeth with necrotic pulp, such as reduced apical size, could hinder the influx of blood and cells to the root canal and possibly negatively impact the outcomes of REP. Moreover, an age-dependent decrease in pulp regeneration using dental pulp stem cells (MDPSCs) due to the reduction of the migration, proliferation, and cell survival of resident stem cells has to be considered [24].

Therefore, the body of knowledge supporting the benefits of Regenerative Endodontics, mainly produced with immature teeth studies, may not directly apply to mature teeth with necrotic pulp. Although contemporary conventional endodontics is assertive in the recovery of mature teeth, there is interest in investigating the challenges and strategies to perform regenerative treatments in adults [4,23]. A recent systematic review of the literature pointed out with moderate certainty the evidence that REP appears to be a viable treatment alternative when compared to conventional endodontic treatment for teeth with closed apex, necrotic pulp, and periapical lesion [25].

However, the comparison between REP and conventional endodontic treatment related to clinical parameters becomes questionable since pulp sensitivity tests would not be applicable to a group with filled canals. An analysis comparing teeth before and after REP, therefore, could contribute to a better understanding of REP with mature teeth with necrotic pulp. In this context, the present study aimed to evaluate clinical and radiographic parameters of mature teeth with necrotic pulp before and after REP through systematic review and meta-analysis. A meta-analysis was performed with the available evidence for REP regarding bone healing and the achievement of a positive response to sensitivity testing that, according to the AAE (2018) [20] protocol, are primary and tertiary objectives of the REP, respectively.

## 2. Materials and Methods

### 2.1. Research Question

This systematic review was conducted and reported following the PRISMA statement [26]. The protocol was registered in the PROSPERO database [27] under the registry CRD42020197364 (University of York, York, UK). Articles were selected to answer the following focused question: “Is Regenerative Endodontics effective for the treatment of mature teeth with necrotic pulp?”.

### 2.2. Eligibility Criteria

Since no comparison was expected with conventional endodontic therapy, this review based the eligibility criteria on a PIO variant of the PICO framework [28], which is adequate to investigate the effectiveness of interventions without a comparator [29]. A structured question was produced, in which: Population (P): permanent dentition (mature teeth); Intervention (I): Regenerative Endodontic Procedures; and Outcomes (O): periapical lesion reduction and/or tooth sensitivity assessment. Two key aspects were considered for the inclusion of studies: (i) clinical study design and (ii) studies on mature teeth with necrotic pulp with closed apex. In this sense, this systematic review included randomized controlled trials (RCT) and clinical studies conducted on permanent mature teeth with necrotic pulp, treated using REP that evaluated periapical lesion reduction and/or tooth sensitivity response.

Even though scientifically relevant, study designs and types of publication that did not produce the type of primary data pertinent to the present analysis were also excluded: in vitro studies, animal studies, studies on immature or open apex teeth, case reports, and literature reviews.

### 2.3. Information Sources

Electronic searches were performed on 9 September 2020 in PubMed, Cochrane Library, Scopus, Web of Science, and Latin American and Caribbean Health Sciences Literature database (LILACS)/Brazilian Library in Dentistry (BBO). The grey literature, defined as documents produced on all levels of government, academics, business, and industry in print and electronic formats but not controlled by commercial publishers, was explored using the databases Bielefeld Academic Search Engine (BASE), Science.gov (accessed on 9 September 2020 (Office of Scientific and Technical Information, Oak Ridge, TN, USA), Global ETD search (Networked Digital Library of Theses and Dissertations, Blacksburg, VA, USA), and OAlster (OCLC, Dublin, CA, USA).

### 2.4. Search Strategy

Specific search strategies were developed for each database (Table S1). No restrictions regarding publication date or language were applied. Terms referring to the population (P) and intervention (I) of the PIO acronym were searched. The terms “regenerative endodontic” AND “permanent dentition” were explored in the grey literature databases.

### 2.5. Study Selection

Two reviewers (P.S. and F.P.G.) independently read the retrieved studies’ titles and abstracts, removed the duplicates, and selected the studies following the eligibility criteria. Full texts were examined to confirm their eligibility. Any disagreement on the suitability of the selected studies was solved through discussion and consensus with a third reviewer (I.P.C.).

### 2.6. Data Extraction

For data extraction, scientific and technological information items were tabulated and analyzed in Microsoft Office Excel 2013 (WA, Redmond, DC, USA). Two reviewers (F.N.S and E.S.L.) performed the analyses independently, with a high level of tested agreement. The extracted data of the selected studies included: author(s) and the year of publication, number of participants, number of teeth, type of teeth, presence of control, regenerative material used, irrigation solution, intracanal medication, size K-file apical to induce bleeding near the apical foramen, internal sealing, external sealing, follow-up period, the type of anesthetic used, the time elapsed from the first visit, and outcomes regarding tooth survival assessment and clinical and image evaluations.

### 2.7. Quality Assessment

The assessment of the methodological quality of all included studies was performed by I.C. and M.Z.S. The methodological quality of the randomized controlled trials (RCT) was assessed using the Revised Cochrane risk-of-bias tool (RoB 2) [30]. For primary RCT studies, the following methodological parameters were recorded, according to the RoB 2 tool: randomization process, deviations from intended interventions, missing outcome data, measurement of the outcome, selection of the reported result, and overall results. The studies were classified as low risk of bias, high risk of bias, or presenting some concerns.

In addition, the methodological quality of the regenerative intervention groups of all studies, including the non-randomized clinical trials, was assessed by the Before-and-After tool (National Heart, Lung, and Blood Institute, Bathesda, MD, USA) [31], considering the outcomes of periapical lesion reduction and tooth sensitivity response after the REP intervention. The Before-and-After tool criteria were used to assess the internal validity of the studies, i.e., to evaluate the extent to which the results reported for REP could be attributed to the intervention and not to bias. Eleven criteria of the Before-and-After tool were applied, except the criterion referring to “group-level intervention”, since the interventions were performed and evaluated at the individual level. The used criteria are described in full with the Results in the “Quality Assessment” section. The answers to each of the questions could be “Yes”, “No”, “Cannot determine”, “Not reported,” and “Not applicable”. The summary quality rating ranged from “Good” (8–11 “Yes” answers) to “Fair” (5–7 “Yes” answers), and “Poor” for 1–4 “Yes” answers [31].

### 2.8. Synthesis of Results

Success was measured according to the attainment of elimination of symptoms, with evidence of bone healing and positive response to sensitivity testing, among the main objectives of REP [20]. To evaluate the prognostic of REP for mature teeth with necrotic pulp through clinical and radiographic parameters, the data were analyzed using the Microsoft Excel add-in MetaXL 5.3 (2019, Version 5.3, EpiGear International, Sunrise Beach, Australia). Analyses of the reports were performed to evaluate the periapical lesion reduction adopting the last follow-up of each study and the 12-month follow-up. Electrical and cold tests were compared after 12 months of treatment. The overall incidence of success of REP was evaluated adopting the last follow-up time of each study.

In all analyses, the number of teeth that presented positive results and the total number of teeth evaluated were included to assess the incidence of therapy success and its 95% confidence interval (CI). A meta-analysis fixed-effect model was applied when a small number of studies were included (three or fewer studies), and a random-effect model was applied when four or more studies were included in the meta-analysis [32]. Heterogeneity was tested using the I^2^ index, and the predictor intervals were calculated in analyses where a random effect was applied.

Seeking harmonization of outcomes across reviews and aiming to generate evidence [30], the authors indirectly compared the meta-analysis results for REP in the treatment of mature teeth with those reported on previous reviews for REP in the treatment of immature teeth with a necrotic pulp [15,21]. The threshold used to compare the results was based on a clinical healing success and periapical lesion reduction rate of 93% (95% CI, 88.16–96.00%), and a radiographic threshold of 88% [21]. Similarly, the threshold of 60% was set for positive sensitivity response to either cold or electrical tests, corresponding to previously reported success rates for immature teeth [15]. In this sense, the meta-analysis pooled the incidences of the outcomes of interest, which were then compared to the bone healing and positive sensitivity response testing thresholds reported to REP in the treatment of immature teeth.

### 2.9. Certainty of Evidence

The Grading Recommendations Assessment, Development and Evaluation framework (GRADE (GRADEpro GDT, 2020, GRADE working group, Hamilton, ON, Canada), available at https://gradepro.org/ accessed on 9 November 2020) [33] was used to systematically assess the certainty of evidence. In this context, before and after clinical trials were initially rated as of low certainty of evidence. However, the quality or certainty of evidence decreased to “very low” if serious or very serious issues were identified related to the risk of bias, imprecision, inconsistency, indirectness, and publication bias [34]. The Indirectness assessment considered the intracanal medication and internal seal material used in REP. The thresholds reported for immature teeth by the reviews of Ong et al. (2020) [21] and Diogenes et al. (2013) [15], described above, were used to assess imprecision, based on the results of Regenerative Endodontics in the literature.

## 3. Results

### 3.1. Study Selection

**Figure 1:**
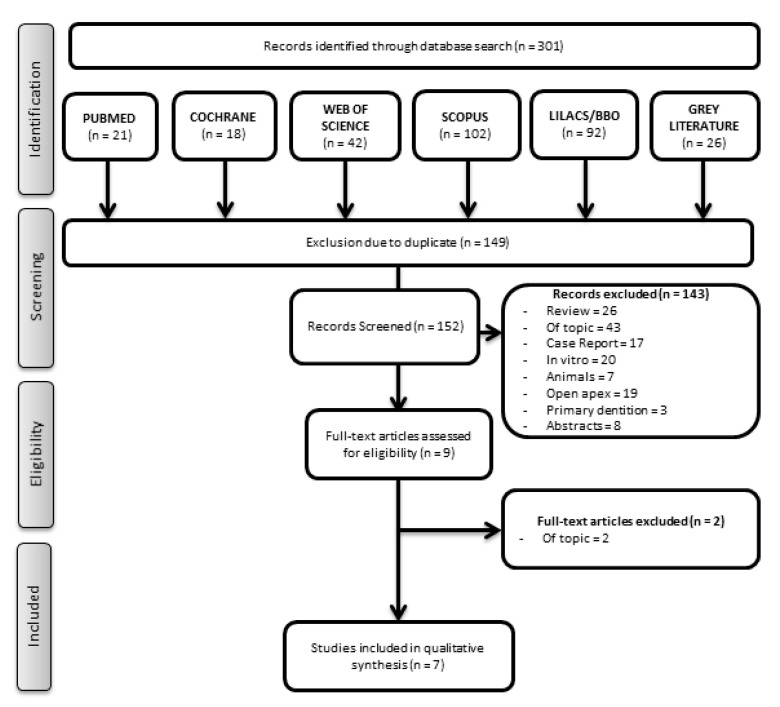
PRISMA flow diagram.

Three hundred and one records were obtained from the search. From those, 149 duplicates were removed, and 152 records remained (Figure 1). Considering the eligibility criteria, 143 studies were excluded: 26 reviews, 43 off-topic, 17 case reports, 20 in vitro studies, 7 animal studies, 19 studies that were performed on permanent teeth with open apex, 3 studies that were conducted on primary dentition, and 8 abstracts. Therefore, during the screening, nine articles [35,36,37,38,39,40,41,42,43] were selected, two of which were excluded [36,42] after evaluating the full texts since they did not evaluate the selected outcomes (Table S2). One was a clinical study that evaluated whether evoked bleeding from the periapical tissues elicited the influx of mesenchymal stem cells (MSCs) into the root canal system in mature teeth with apical lesions [36], and the other aimed exclusively to evaluate tooth color changes [42]. Consequently, the screening of records identified seven studies meeting all of the inclusion criteria. All selected papers were written in English, although the language was not limited in the search.

### 3.2. Main Characteristics of the Included Articles

The primary data for each selected paper are summarized in Table 1. Of the seven included studies, four were randomized controlled trials [39,40,41,43], and three were non-controlled clinical studies [35,37,38]. Considering the biological material used for the REP, most of the studies used blood clots, also called SealBio [35,38,39,40,43], while two studies used platelet aggregates [38,41].

### 3.3. Quality Assessment

Regarding the methodological quality of the four RCT studies, the study conducted by Arslan et al. (2019) [39] had a low risk of bias, while Jha et al. (2019) [40], El-Kateb et al. (2020) [43], and Brizuela et al. (2020) [41] had some concerns according to the criteria of Cochrane risk-of-bias tool for randomized trials (RoB 2), as described in Table 2. The limitations observed referred to randomization and deviations from intended interventions [40] and the selection of the reported results [43]. Brizuela et al. (2020) [41] had a high risk of measuring the outcomes and some concerns regarding selecting reported results. These limitations will be further examined in the Discussion section.

The Before-And-After quality assessment was applied to all selected papers, analyzing 11 items regarding the REP groups. A single study was classified as having poor methodological quality [35], two were considered fair [37,38], and four were considered to have good methodological quality regarding the regenerative intervention [39,40,41,43] (Table S3). Regarding the quality criteria, it was observed that the main concerns were related to the sample size, which was not sufficiently large to provide confidence in the findings [35,37,38,39,40,41]. Besides, most of the studies lacked clarity about the inclusion of all eligible participants [35,37,38,40,41].

### 3.4. Synthesis of Results

A meta-analysis was performed by evaluating the prognosis of REP in mature teeth with necrotic pulp. The incidence of events (success, reduction of periapical lesion, response to electrical and cold tests) was evaluated after REP, compared to before treatment. Therefore, it was possible to include both RCTs and non-randomized studies with parallel or single-arm (before and after) designs in the same meta-analysis since only the comparable REP groups were included in the present quantitative analysis.

Data exclusively related to the REP intervention in all included studies were statistically analyzed and interpreted to integrate the reported information, considering the overall success incidence of mature teeth treated with REP, reduction of periapical lesion obtained after REP, and electrical and cold test responses after REP, as described below. The summary of the certainty of the evidence for each comparison, evaluated using the GRADE approach, is presented in Table 3.

#### 3.4.1. Success Incidence

**Figure 2:**
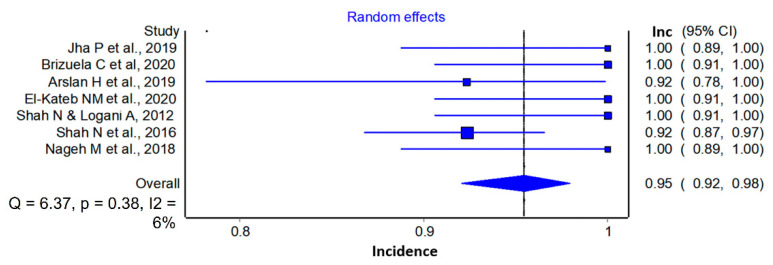
Incidence of success of mature teeth with necrotic pulp treated with REP.

Seven studies [35,37,38,39,40,41,43] were included in this analysis. Of 228 mature teeth with necrotic pulp treated with REP, 217 could be considered successful (0.95 (0.92, 0.98) I^2^ = 6%) (Figure 2) with a prediction interval range from 0.84 to 0.98, in comparison to the previously estimated results for REP in immature teeth [21]. According to the GRADE framework, the certainty of the evidence was classified as very low (Table 3).

#### 3.4.2. Reduction of Periapical Lesion

**Figure 3:**
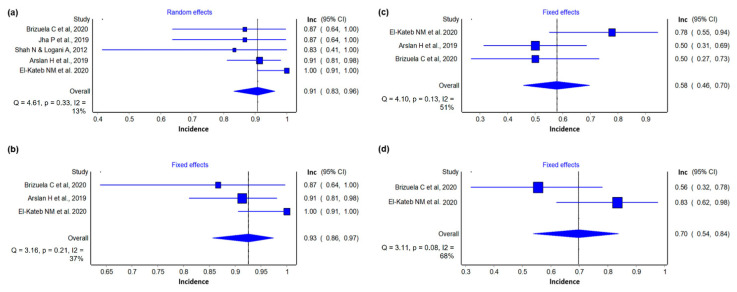
Outcomes of meta-analysis at: (**a**) incidence of periapical lesion reduction of mature teeth with necrotic pulp treated with REP; (**b**) incidence of periapical lesion reduction of mature teeth with necrotic pulp treated with REP with 12-month follow-up; (**c**) incidence of positive electrical test response of mature teeth with necrotic pulp treated with REP with 12-month follow-up; (**d**) incidence of positive cold test response of mature teeth with necrotic pulp treated with REP with 12-month follow-up.

Five studies [35,39,40,41,43] were included in this analysis. Considering the last period of follow-up studies of 100 mature teeth with necrotic pulp submitted to REP, 91 were considered successful (0.91 (0.83, 0.96) I^2^ = 13%) (Figure 3a), with a prediction interval range from 0.70 to 0.97, in comparison to the estimated results for REP in immature teeth [21].

Considering a 12-month follow-up of 79 mature teeth with necrotic pulp treated with regenerative endodontics, 73 treatments were considered successful (0.93 (0.86, 0.96) I^2^ = 37%), in comparison to the estimated results for REP in immature teeth [21] (Figure 3b). The certainty of evidence assessed by GRADE was classified as very low (Table 3).

#### 3.4.3. Electrical Test

Three studies [39,41,43] were included in this analysis. Of 64 mature teeth with necrotic pulp treated with REP, 37 presented a positive response to the electrical pulp test (0.58 (0.46, 0.70) I^2^ = 51%) (Figure 3c). The GRADE framework’s certainty of the evidence was very low (Table 3).

#### 3.4.4. Cold Test

Two studies [41,43] were included in this analysis. Of 36 mature teeth with necrotic pulp treated with REP, 25 presented a positive response to the cold pulp test (0.70 (0.54, 0.84) I^2^ = 68%) (Figure 3d). The certainty of evidence according to the GRADE framework was very low (Table 3).

## 4. Discussion

The present study selected primary articles for a systematic review whose outcomes led to a meta-analysis. In the following sessions, the main characteristics of the studies will be discussed with regard to important methodological aspects of the REP. Subsequently, the evidence for the success of these treatments used for mature teeth with necrotic pulp will be discussed in light of the meta-analysis results.

### 4.1. Patients’ Age and REP

The studies included in the present review showed a wide variation in their age groups, which did not allow the assessment of correlations between age and the success of REP on mature teeth with necrotic pulps. A previous study on immature teeth reported that younger patients (9–13 years) obtained better results in the REP than older participants (14–18 years old) [44]. Regarding mature teeth with necrotic pulp, a single study assessed the effect of age on REP. Arslan et al. [39] performed a regressive analysis of confounding variables, concluding that age had no significant effect on the healing size of the radiographic lesion. Therefore, even though the results by Arslan et al. [39] may provide initial evidence of the independence of age for the success of REP in mature necrotic teeth, this claim still needs support from further assessments through clinical studies with an increased number of participants and age groups.

### 4.2. Management of Blood Clot and Derivatives

The use of apical enlargement in REPs seeks to form a vascularized tissue similar to the pulp tissue through the blood influx from the periapical tissues. Therefore, it allows for the structuring of an autologous scaffold containing growth factors, contributing to the migration of mesenchymal cells [36]. Such procedures have obtained promising results in immature teeth due to the presence of such cells in periapical tissues [45,46].

Similarly, Chrepa et al. (2015) [36] showed that periapical over-instrumentation in mature teeth provides blood and undifferentiated mesenchymal cell influx. For this purpose, the use of an anesthetic without a vasoconstrictor on the second visit was essential since it could facilitate the blood supply that acts as a reservoir of cells and growth factors [47], as proposed in the protocols employed by Nageh et al. (2018) [38], Arslan et al. (2019) [39], Jha et al. (2019) [40], and El Kateb et al. (2020) [43].

Bleeding can be optimized with an intentional over-instrumentation 2–3 mm past the apex into the periapical region, using K-files #20–#40 to induce it near the apical foramen [35,37,38,39,40,43]. It is worth mentioning that the final diameter of the apical foramen should allow the migration of existing cells in the periodontal ligament. The tip of the instrument (D0) used to evoke bleeding in most studies [35,37,38,39,40,43] ranged from 0.2 to 0.4 millimeters (200–400 μm). Interestingly, a review [5] concluded that clinical success was achieved after REP in mature teeth with necrotic pulp with an apical diameter size <1.0 mm. In this sense, considering the dimensions of cementoblasts and osteoblasts (10–100 μm) [44] and the D0 of the instruments used, the influx of these cells through the apical foramen could be accomplished, favoring the REP.

Blood derivatives such as platelet aggregates have been widely used in dentistry for regenerative procedures in soft and hard tissues due to their fibrin composition capable of sustained release of growth factors and inflammatory cytokines [48]. The main autologous blood processing protocols documented in the literature are PRP and PRF, which allow the production of bioactive materials capable of acting as scaffolds for cell migration and proliferation [49]. They have been widely studied in different approaches of regenerative dentistry, including their combination with autogenous bone or substitutes as graft materials before dental implant rehabilitation since they contribute to increased cell proliferation, osteoblasts differentiation, mineralization, and neovascularization [47].

Two selected studies employed autologous blood processing protocols for REP. Brizuela et al. (2020) [41] employed mesenchymal stem cells from the umbilical cord (UC-MSCs) encapsulated into a scaffold consisting of platelet-poor plasma (PPP), derived from the protocol of production of PRP. This first-generation platelet concentrate consists of two sequential centrifugations of blood aliquots collected in the presence of an anticoagulant and later addition of thrombin and/or calcium chloride, producing a network of fibrin (PRP) and acellular plasma (PPP). In this context, PRP can promote neoangiogenesis, cell proliferation and differentiation, and control of the local inflammatory process due to its content of both pro and anti-inflammatory cytokines, such as interleukins 1, 6, and 10, and growth factors such as TGF-β, platelet-derived growth factor, and vascular endothelial growth factor, resulting in improved wound healing, and even acting as an “immunological nodule” [50].

It is noteworthy that, in this case, these materials were of heterologous origin, with cells originated from a cell bank (Cells for Cells, Santiago, Chile) and PPP fractions from the irradiated plasma of healthy donors. Although the use of heterologous materials is considered a viable alternative, the cost of the procedure and the risk of cross-contamination are both increased [51].

The second study, by Zhou et al. (2017) [52], compared the use of isolated blood clots and those associated with platelet-rich fibrin (PRF) in REP’s performance. PRF is a second-generation platelet aggregate with a simplified protocol proposed by Dohan et al. (2006) based on single centrifugation (400× *g* for 10 min) of blood aliquots without the addition of anticoagulants or any other substance, in a low-cost method. This protocol allows the production of an autologous biomaterial through the entrapment of platelets, immune cells, and growth factors in its fibrin network, providing structural stability and long-term production/release of growth factors [48]. Nevertheless, while both protocols were satisfactory, the authors identified no benefits regarding the inclusion of the PRF in the blood clot [52].

### 4.3. Root Canal Irrigants

Another point to be highlighted in the included studies was the recurrent use of ethylenediaminetetraacetic acid (EDTA) [38,39,40,41,43]. The usefulness of EDTA in REP as an intracanal irrigant has already been reported [53]. Also, Pang et al. (2014) [54] showed that EDTA treatment of the dentin surface could promote odontoblast/osteoblast differentiation of the attached cells. EDTA promotes stem cell survival and intimate adhesion on the dentin [55], most probably due to the release of growth factors from the EDTA-treated dentin matrix. These may include tumor growth factor–β, bone morphogenic protein–2, platelet-derived growth factor, and vascular endothelial growth factor [56,57,58]. The fact that the majority of the studies identified in this search employed EDTA as an irrigant most probably reflects a consensus in the field, reinforced by the fact that it is proposed in the main harmonized protocols of REP, such as AAE (2018) [20] and the position statement by the European Society of Endodontics [22].

### 4.4. Root Canal Medication

Regarding the intracanal medications used in the REP protocols of the included studies, diverse substances were employed. While both antimicrobial materials for irrigation and intracanal medications have limitations [59], a significant reduction in the root canal microbiota is a key aspect of the REP’s success. Their wide use in REP occurs mainly due to antibiotic pastes’ ability to eradicate bacteria present in dentinal tubules [60].

However, some relevant studies have shown that concentrations equal to or greater than 1 mg/mL of antibiotic pastes can produce adverse effects on stem cells of the apical papilla (SCAPs). On the other hand, calcium hydroxide promotes the proliferation of SCAPs, possibly through the release of bioactive growth factors from dentin [61]. Therefore, the AAE recommends using both tri-antibiotic pastes or calcium hydroxide as intracanal drugs [4], even though the use of antibiotic paste formulations alone is most common [62], as confirmed by the selected studies in this review [35,37,38,39,40]. The diversity of medications used in the different protocols may be a limiting factor for a definitive conclusion about their performance in the REP outcomes for mature teeth with necrotic pulp. Interestingly, none of the included studies in the present review mentioned intracanal calcification during the follow-up period. Conversely, the literature has shown that REP could result in this undesirable effect with total or partial pulpal obliteration in immature teeth due to multiple contributing factors, such as the type of medication used and the induction of intracanal bleeding [63].

### 4.5. Quality Assessment

Randomized controlled trials are considered a reference of excellence or a gold standard among all methods of clinical investigation. They provide direct scientific evidence with less probability of error to clarify a cause–effect relationship between two events. In this sense, this review included four RCT studies, even though three of them presented some methodological concerns [40,41,43], and just one had a low risk of bias [39], according to the Rob 2 tool (Higgins et al., 2019).

The limitations observed in Jha et al. (2019) [40] referred to randomization since there is no information about concealment of the allocation sequence and deviations from intended interventions. El-Kateb et al. (2020) [43] had some concerns on the selection of the reported results since the magnetic resonance imaging used to assess pulp tissue regeneration has limitations assumed by the author of the primary study due to the production of artifacts by metallic devices or even by the movement of the patient due to swallowing or anxiety. Brizuela et al. (2020) [41] had a high risk of measuring the outcomes: even though it used a validated method (Laser Doppler flowmetry) to measure the vitality of the REP treated teeth, the sensitivity test results were compared to the control group that underwent conventional endodontic treatment. This comparison would be inappropriate for this outcome since no vitality was expected for such teeth. The authors considered the report of a positive response for sensitivity tests for necrotic teeth at a basal time, which was not clearly defined, and after conventional treatment as suggestive of selection bias.

Since the present systematic review did not intend to compare REP with conventional endodontic treatments but to assess the outcomes of REP interventions, non-RCT before-and-after studies were also included. Considering the outcomes of interest (periapical lesion reduction and sensitivity response) as the most relevant for REP, all the studies were also evaluated by Before-And-After criteria, regardless of study design [31,64]. Interestingly, the studies rated with good methodological quality [39,40,41,43] presented an RCT design in accordance with the concept of the more detailed and rigorous methodology protocols of this type of study design. However, regarding the Before-And-After criteria, it could be suggested that larger and more representative samples could contribute to the higher evidence regarding REP for mature teeth with necrotic pulp, a factor to be considered in future studies.

### 4.6. Outcomes

Regarding the outcomes and assessing methods, some authors considered that the treatment success was restricted to the absence of symptoms and the reduction or resolution of the periapical radiolucency [35,37,40]. On the other hand, other studies had a broader perspective, assessing the clinical response through sensitivity and vitality tests besides the periapical lesion’s resolution [38,39,41,43]. Considering the definition of REP, it could be assumed that studies that did not apply such tests could not produce conclusive results concerning the replacement of vital tissue. However, despite the distinct methodologies used to assess the outcomes, an overall comparison of the success incidence could be performed to contribute to the understanding of REP in treating mature teeth with necrotic pulp. Interestingly, this analysis pointed out a high incidence of success not so discrepant to the overall healing success rate of REP reported in immature teeth with necrotic pulp (93% (95% CI, 88.16–96.00%)) [21]. Likewise, the separate analysis of the periapical lesion reduction revealed a high success incidence, both considering the last period of follow-up of five included studies [35,39,40,41,43] and the 12-month follow-up period [39,41,43], once again not distinct from previous reports of success rate for immature teeth healing through REP [21].

Moreover, the analysis of the results of cold [41,43] and electrical tests [38,39,41,43] disclosed that more than a half of the mature teeth treated with REP presented positive responses to the cold test, with slightly reduced positive responses obtained by the electrical test [39,41,43] at the 12-month follow-up period. It is important to notice that the sensitivity results identified in this review for mature teeth with necrotic pulp were consonant with those previously reported for immature teeth with necrotic pulp after REP [15].

The overall results of this systematic review identified evidence indicating REP as successful in the treatment of mature teeth with necrotic pulp, suggesting the clinical relevance of REP as a paradigm shift in conventional endodontics. It is noteworthy that the use of biological approaches (blood clot and derivatives) offers the opportunity to regain the sensitivity to thermal and electrical tests, even in mature teeth. A regenerated dental pulp restores the flow of immunological cells to the innate immunity within the canal root, reducing potential reinfections, increasing teeth hydration, and increasing the mechanical resistance to fractures, as compared to endodontically treated teeth [4].

Among the present review’s limitations, the small number of articles is included, as the search was restricted to mature teeth with closed apex, and the impossibility of performing some statistical comparisons due to the high methodological heterogeneity of the selected studies. These restrictions limit the conclusions of the present review as rather preliminary, and it is patent that more high-quality clinical studies are still needed to support the statement that REP is related to a good prognosis in mature teeth, mainly due to the overall low certainty of evidence available to date. The identified factors that most influenced the downgrade of the certainty of the evidence were the risk of bias, the heterogeneity, and the indirectness (external validity). Considering the outcomes of interest, the periapical tissues repair could be clearly assessed and compared by the radiographic parameters, observing periapical lesion reduction. However, difficulties regarding the evaluation of the pulp health status were observed, as this remains a challenging subject [65]. The thermal test is rather subjective, as it depends on the pain threshold of participants, and the selected studies did not describe methodological details for its performance. Furthermore, only two studies presented results for the comparison, contributing to high heterogeneity. The electrical sensitivity tests were used to indirectly measure the pulp condition in three studies, two of which described the use of the same device, but only one presented the parameters considered as a cutoff for a vital pulp [43]. Therefore, it is possible that the three studies reporting electrical tests may have employed conflicting parameters to suggest nonvital teeth, and there is not a normal value for pulp test readings, increasing the risk of high heterogeneity related to methodological differences and individual variability. It is worth mentioning that the Laser Doppler flowmetry, employed by Brizuela et al. [41], seems to be the most accurate, painless, and objective method to assess the pulp health status, allowing to directly evaluate the tissue blood flow [65].

In this sense, the authors recommend the proposal of a standardized REP clinical protocol designed to mature teeth with necrotic pulp, based on the best evidence available, that could support future studies with good methodological quality. The best level of evidence could come from randomized controlled clinical trials whose controls were vital homologous teeth and not conventional endodontically treated teeth, as these would not respond to thermal and electrical tests. Further clinical studies should also consider larger sample sizes and extended follow-up periods to increase validity, and special care on randomization, measurement of outcomes (and their methodological reports), and selection of reported results to reduce risk of bias and achieve high certainty of the evidence on the success of REP for mature teeth.

## 5. Conclusions

There is a high incidence of success of REP on the healing of mature teeth with a necrotic pulp, evidenced by reduction on the periapical lesion and positive responses to sensitivity tests. However, the low certainty of the available evidence indicates the need for more clinical studies to determine the clinical relevance of Regenerative Endodontic Procedures as a paradigm shift in conventional endodontics.

## Figures and Tables

**Table 1:** Main characteristics of the selected articles.

Author/Year of Publication	N. of Participants	Tooth	Control	Regerating Material	Irrigating Solution	Intracanal Medication	Apical File	Internal Sealing	External Sealing	Follow-Up	At 2nd Visit		Outcome
											Anesthetic	Period	
Brizuela C et al., 2020 ^a^	36 11♂ 25♀ (16–58 y)	Incisors, canines, mandibular premolars	Guta percha conventional obturation	PPP umbilical cord mesenchymal stem cells	2.5% NaOCl EDTA	Calcium hydroxide	#8 K-file (bleeding)	Absorbable gelatin sponge hemostats (Gelita-Spon^®^ GmbH, Eberbach, Germany) and Biodentine (Septodont, France)	Resin (Filtek™ Z350 XT Universal Restorative; 3M ESPE, St Paul, MN, USA)	6 and 12 months	Not reported	3 weeks	Radiography and Cone-beam computed tomography (CBCT). Vitality by Laser Doppler Flowmetry (LDF) and the Perfusion Unit (PU) percentage. Cold Test: 56% were responsive. Hot test: 28% were responsive. Eletric Test: 50% responsive. Cortical compromise: 89% not present.
El-Kateb NM et al., 2020 ^a^	18 (20–34 y)	17 maxillary central incisors and 1 lateral incisor	X5 Blood clot	X3 Blood clot	1.5% NaOCl 17% EDTA	Ultracal XS calcium hydroxide (Ultradent Products GmbH, South Jordan, UT)	Widening Protaper next X3 and Protaper next X5 K-file #25 (bleeding)	Biodentine (Septodont, Saint-Maur-des-Fosses, France)	GIC + resin composite	1, 3, 6, 9, and 12 months	3% mepivacaine without vasoconstrictor	Not reported	Clinical evaluation, digital radiographs, magnetic resonance imaging (MRI), sensitivity test (cold test and electrical test). 60% of the cases regained sensitivity after 12 months.
Arslan H et al., 2019 ^a^	46 35♂ 11♀ (18–30 y)	32 maxillary anterior teeth + 16 mandibular anterior teeth	Guta percha+ epoxy resin– based sealer (2Seal; VDW, Munich, Germany)	Blood clot	1% NaOCl 2 mL 5% EDTA distilled water	Triple antibiotic paste (doxycycline, metronidazole, and ciprofloxacin)	K-file #25 (bleeding)	MTA	MTA + resin composite material (Universal Restorative 200, 3M ESPE)	12 months	Isocaine 3%; (Novocol, ON, Canada)	3 weeks	Clinical and radiograph evaluation, EletricalTest. 92.3% successful cases and 50% positive response to EPT in REP.
Jha P et al., 2019^ a^	30 (9–15 y)	30	Guta percha conventional obturation	SealBio	2.5% NaOCl EDTA	Triple antibiotic paste (ciprofloxacin, metronidazole and minocycline)	Widening K-files #25–#30 #20 K-file (bleeding)	Calcium sulfate-based cement (Cavit G)	Not reported	6, 12, and 18 months	3% mepivacaine without adrenaline	1 or 2 weeks	Clinical and radiographic evaluation. 86.66% of the teeth were considered completely cured in group I (SealBio) and 80% in group II (obturation).
Nageh M et al., 2018 ^b^	15 (18–40 y)	Maxillary central incisor	No	PRF e Blood clot	1.5% NaOCl 17% EDTA	Double antibiotic paste (DAP) (metronidazol, ciprofloxacin)	K-files #20–#40 (Widening and bleeding)	MTA	GIC base and resin composite	Every 3 months for a follow-up of 1 year	3% mepivacaine	3 weeks	Thermal (cold) and electrical tests were used. 60% of the teeth had vital pulp and 40% partial vitality after 12 months.
Shah N, 2016 ^b^	116 76♂ 40♀ (12–80 y)	134	No	SealBio	2.5% NaOCl. Final wash with Betadine	Triple antibiotic paste (ciprofloxacin, metronidazole, tetracycline) or calcium hydroxide	Widening K-files #25 K-files #20 (bleeding)	Calcium sulfate-based cement (Cavit, 3M ESPE USA)	Silver amalgam/ composite/and full coverage coronal restoration	Every 6 months for a follow-up of 6 years	Not reported	5–7 days	Clinical and radiographic evaluation. 16 cases were lost to follow-up. Approximately 97% of cases treated with the new technique were successful.
Shah N and Logani A 2012 ^b^	18 11♂ 7♀ (15–76 y)	Not reported	No	SealBio	2.5% NaOCl	Triple antibiotic paste (metrogyl, ciprofloxacin and tetracycline)	Widening K-files #25-#30 K-files #20(bleeding)	Calcium sulfate-based cement (Cavit, 3M ESPE USA)	Not reported	Every 6 months for a follow-up of 3 years	Not reported	Not reported	Lesion size, bone, and cementum density in HU and periapical index (CBCT-PAI). Remarkable decrease in the lesion size and increase in bone and cementum density were documented.

^a^ randomized controlled trials, ^b^ non-controlled clinical studies.

**Table 2:** Quality assessment of the randomized clinical trials according to the RoB 2 tool.

Scheme 2020.	Randomization Process	Deviations from Intended Interventions	Missing Outcome Data	Measurement of the Outcome	Selection of the Reported Result	Overall
**Brizuela et al. 2020 [37]**	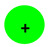	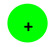	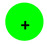	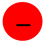	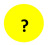	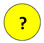
**El-Kateb et al. 2020 [39]**	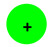	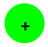	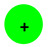	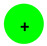	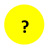	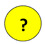
**Arslan et al. 2019 [35]**	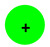	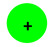	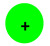	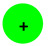	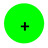	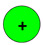
**Jha et al. 2019 [36]**	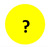	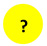	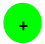	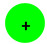	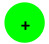	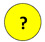
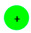 Low risk 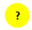 Some concerns 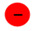 High risk						

**Table 3:** Assessment of the certainty of evidence according to GRADE.

Certainty Assessment								Certainty
No. of Studies	Study Design	Risk of Bias	Inconsistency	Indirectness	Imprecision	Other Considerations	No. of Mature Necrotic Teeth	
**Success**								
7	Before and after	Serious ^a^	Serious ^b^	Not serious	Not serious	None	228	⨁◯◯◯ VERY LOW
**Reduction of periapical lesion (last follow-up)**								
5	Before and after	Serious ^a^	Not serious	Not serious	Serious ^c^	None	100	⨁◯◯◯ VERY LOW
**Reduction of periapical lesion (12 months)**								
3	Before and after	Serious ^a^	Serious ^b^	Serious ^d^	Serious ^c^	None	79	⨁◯◯◯ VERY LOW
**Electrical pulp test**								
3	Before and after	Serious ^a^	Very serious ^b^^,^^e^	Serious ^d^	Serious ^c^	None	64	⨁◯◯◯ VERY LOW
**Cold pulp test**								
2	Before and after	Serious ^a^	Very serious ^b^^,^^e^	Serious ^f^	Serious ^c^	None	36	⨁◯◯◯ VERY LOW

^a^ Most of studies presented some type of risk of bias. ^b^ Some variability in point estimates. ^c^ Lower limit of confidence interval is below threshold. ^d^ Results could not be extrapolated to internal seal. ^e^ Substantial or considerable heterogeneity. ^f^ Results could not be extrapolated to intracanal medication and internal seal.

## Data Availability

Data sharing not applicable.

## Supplementary Materials

The following are available online at https://www.mdpi.com/article/10.3390/ma14164418/s1, Table S1: Electronic Database and Search Strategy. (9 September 2020), Table S2: Full text evaluated and excluded from systematic review, Table S3: Quality Assessment Tool for Before–After (Pre–Post) Studies.
