# Supplementary material for: Prognosis of Regenerative Endodontic Procedures in Mature Teeth: A Systematic Review and Meta-Analysis of Clinical and Radiographic Parameters
Source: Materials (Basel). 2021 Aug 6;14(16):4418. doi: 10.3390/ma14164418 (PMC8398537; doi:10.3390/ma14164418)
Supplement: Supplementary file 1 [file materials-14-04418-s001.zip › REP - Table S1.pdf]

Prognosis of regenerative endodontic procedures in mature teeth: a systematic review and meta-analysis of clinical and radiographic parameters

Table S1. Electronic Database and Search Strategy

**PUBMED**	
#1 Dentition, Permanent[MeSH Terms]) OR Dentition, Secondary[Title/Abstract]) OR Dentition, Adult[Title/Abstract]) OR "Mature teeth"[Title/Abstract]) OR "Mature tooth"[Title/Abstract]	#2 Regenerative Endodontics[MeSH Terms]) OR Endodontic, Regenerative[Title/Abstract]) OR "Regenerative Medicine"[Title/Abstract]) OR Revitalization[Title/Abstract]
**#1 AND #2**	
**SCOPUS**	
#1 (TITLE-ABS KEY (dentition, AND permanent) OR TITLE-ABS-KEY (dentition, AND secondary) OR TITLE-ABS-KEY (dentition, AND adult) OR TITLE-ABS-KEY (mature AND teeth) OR TITLE-ABS-KEY (mature AND tooth)	#2 (TITLE-ABS-KEY (regenerative AND endodontics) OR TITLE-ABS-KEY (endodontic, AND regenerative) OR TITLE-ABS-KEY (regenerative AND medicine) OR TITLE-ABS-KEY (revitalization)
**#1AND #2**	
**WEB OF SCIENCE**	
#1 **TÍTULO:**(Dentition, Permanent) OR **TÓPICO:**(Dentition, Secondary) *OR* **TÓPICO:**(Dentition, Adult) *OR* **TÓPICO:** (Mature teeth) OR **TÓPICO:** (Mature tooth)	#2 **TÍTULO:**(Regenerative Endodontics) *OR* **TÓPICO:** (Endodontic, Regenerative) *OR* **TÓPICO:** (Regenerative Medicine) *OR* **TÓPICO:** (Revitalization)
**#1 AND #2**	
**LILACS/BBO**	
#1 mh:(Dentition, Permanent)) OR (tw:(Dentition, Secondary)) OR (tw:(Dentition, Adult)) OR (tw:(Mature tooth)) OR (tw:(Mature teeth))	#2 (mh:(Regenerative Endodontics)) OR (tw:(Endodontic, Regenerative)) OR (tw:(Regenerative Medicine)) OR (tw:(Revitalization))
**#1 AND #2**	
**COCHRANE LIBRARY**	
#1 Dentition, Permanent<br>#2 (Dentition, Secondary):ti,ab,kw OR (Dentition, Adult):ti,ab,kw OR (Mature teeth):ti,ab,kw OR (Mature tooth):ti,ab,kw	#3 Regenerative Endodontics<br>#4 (Endodontic, Regenerative):ti,ab,kw OR (Regenerative Medicine):ti,ab,kw OR (Revitalization):ti,ab,kw<br>#5 #1 OR #2<br>#6 #3 OR #4<br>#7 #5 AND #6
