# Supplementary material for: Prognosis of Regenerative Endodontic Procedures in Mature Teeth: A Systematic Review and Meta-Analysis of Clinical and Radiographic Parameters
Source: Materials (Basel). 2021 Aug 6;14(16):4418. doi: 10.3390/ma14164418 (PMC8398537; doi:10.3390/ma14164418)
Supplement: Supplementary file 1 [file materials-14-04418-s001.zip › REP - Table S2.pdf]

Prognosis of regenerative endodontic procedures in mature teeth: a systematic review and meta-analysis of clinical and radiographic parameters

Table S2. Full text evaluated and excluded from systematic review.

Author	Reference	Year	Open Apex	Primary dentition	In vitro	Animal	Case Reports	Review	Of Topic	Cochrane Abstracts	Select
**Gioventù S, Andriolo G, Bonino F, Frasca S, Lazzari L, Montelatici E *et al.***	A novel method for banking dental pulp stem cells. *Transfus Apher Sci* **47**(2), 199-206	2012							X		
**Shah N**	A regeneration-based, nonobturation root-canal treatment for fully-mature teeth: Six years experience with »sealBio». Contemporary Clinical Dentistry **7**(3), 296-301	2016									X
**Jha P, Virdi MS, Nain S.**	A Regenerative Approach for Root Canal Treatment of Mature Permanent Teeth: Comparative Evaluation with 18 Months Follow-up. *Int J Clin Pediatr Dent* **12**(3), 182–188	2019									X
**Bucchi C, Gimeno-Sandig A., Valdivia-Gandur I, Manzanares-Céspedes C, De Anta JM.**	A Regenerative Endodontic Approach in Mature Ferret Teeth Using Rodent Preameloblast-conditioned Medium. *In Vivo* **33**(4),1143-1150.	2019				X					
**Kahler B, Chugal N, Lin LM**	Alkaline materials and regenerative endodontics: A review. *Materials* **10**(12),1389.	2017						X			
**Cordero CB, Santander GM, González DU, Quezada A, Silva CI, Vásquez C *et al.***	Allogeneic Cellular Therapy in a Mature Tooth with Apical Periodontitis and Accidental Root Perforation: A Case Report. *Journal of Endodontics* **46** (12), 1920-1927.e1	2020					X				
**Kahler B, Rossi-Fedele G, Chugal N, Lin LM**	An Evidence-based Review of the Efficacy of Treatment Approaches for Immature Permanent Teeth with Pulp Necrosis. *Journal of Endodontics* **43**(7), 1052-1057.	2017	X								
**Jacobs JC, Troxel A, Ehrlich Y, Gregory RL, Yassen GH *et al.***	Antibacterial Effects of Antimicrobials Used in Regenerative Endodontics against Biofilm Bacteria Obtained from Mature and Immature Teeth with Necrotic Pulps. *Journal of Endodontics* **43(**4),575-579	2017							X		
**Guadarrama PO, Guadarrama QLJ, Robles B, Norma L**	Aplicaciones odontológicas de las células madre pulpares de dientes temporales y permanentes: revisión de estudios in vivo/ Dental applications of pulpal stem cells of temporary and permanent teeth: review of in vivo studies. *Revista ADM* **75** (3),127-134.	2018						X			
**Nageh M, Ahmed GM, El-baz AA**	Assessment of Regaining Pulp Sensibility in Mature Necrotic Teeth Using a Modified Revascularization Technique with Platelet-rich Fibrin: A Clinical Study. *J Endod* **44**(10),1526–1533.	2018									X
**NCT04158232**	Assessment of Regenerative Potential of Mature Permanent Teeth With Necrotic Pulps Using Two Revascularization Protocols. (In Vivo Study)	2020								X	

Continue

Author	Reference	Year	Open Apex	Primary dentition	In vitro	Animal	Case Reports	Review	Of Topic	Cochrane Abstracts	Select
**Del Fabbro M, Lolato A, Bucchi C, Taschieri S, Weinstein RL**	Autologous Platelet Concentrates for Pulp and Dentin Regeneration: A Literature Review of Animal Studies. *Journal of Endodontics* **42**(2), 250-257	2016						X			
**Jung IY, Lee SJ, Hargreaves KM,**	Biologically Based Treatment of Immature Permanent Teeth with Pulpal Necrosis: A Case Series. *Texas dental journal* **129**(6), 601-616	2012					X				
**Raddall G, Mello I, Leung BM**	Biomaterials and Scaffold Design Strategies for Regenerative Endodontic Therapy. *Frontiers in Bioengineering and Biotechnology* **7**.	2019							X		
**Bagú L, Barbich M**	Bone formation from dental pulp stem cells [Article@Formación de tejido óseo a partir de células madre de pulpa dental]. *Actualizaciones en Osteologia* **11**(3), 220-226	2015						X			
**Brizuela C, Meza G, Urrejola D, Quezada MA, Concha G, Ramírez V, et al.**	Cell-Based Regenerative Endodontics for Treatment of Periapical Lesions: A Randomized, Controlled Phase I/II Clinical Trial. *Journal of Dental Research,* **99**(5), pp. 523-529	2020									X
**Maria OM, Khosravi R, Mezey E, Tran SD**	Cells from bone marrow that evolve into oral tissues and their clinical applications. Oral Diseases **13**(1), 11-16	2007						X			
**Wen Q, Jing J, Han X, Ho TV, Cha, Y.**	Cellular and molecular mechanisms of tooth root development. **35**(11), 2252-2264	2020						X			
**Yazid F, Luchman NA, Wahab RMA, Ariffin SHZ**	Characterization and osteogenic differentiation of human dental pulp stem cells and stem cells from exfoliated deciduous teeth [Article@Pencirian dan pembezaan osteogenik sel stem pulpa gigi kekal dan gigi desiduos manusia*]. Sains Malaysiana* **48**(7),1483-1490	2019			X						
**TCTR20161221002**	Clinical evaluation of regenerative endodontic treatment success in mature teeth with periapical lesion	2016								X	
**Chrepa V, Joon R, Austah O, Diogenes A, Hargreaves KM, Ezeldeen M *et al.***	Clinical Outcomes of Immature Teeth Treated with Regenerative Endodontic Procedures-A San Antonio Study. *Journal of Endodontics* **46** (8), 1074-1084.	2020	X								
**Wang H, Zhong Q, Yang T, Qi Y, Fu M, Yang X *et al.***	Comparative characterization of SHED and DPSCs during extended cultivation in vitro. *Mol Med Rep* **17**(5), 6551-6559	2018			X						
**Wang X, Sha XJ, Li GH, Yang FS, Ji K, Wen LY *et al.***	Comparative characterization of stem cells from human exfoliated deciduous teeth and dental pulp stem cells. *Arch Oral Biol* **57**(9), 1231-1240	2012			X						
**Rasha AR, Amr EAEL, Norhan AEWED**	Comparative Study between Revitalization of Necrotic Immature Permanent Anterior Teeth with and without Platelet Rich Fibrin: A Randomized Controlled Trial. *J Clin Pediatr Dent* **43** (2), 78–85.	2019	X								
**Nakajima K, Kunimatsu R, Ando K,**	Comparison of the bone regeneration ability between stem cells from human exfoliated deciduous teeth, human dental	2018			X						

Continue

Author	Reference	Year	Open Apex	Primary dentition	In vitro	Animal	Case Reports	Review	Of Topic	Cochrane Abstracts	Select
**Ando T, Hayashi Y, Kihara T *et al.***	pulp stem cells and human bone marrow mesenchymal stem cells. *Biochem Biophys Res Commun* **497**(3), 876-882.										
**Shah N, Jadhav GR, Mitta P, Logan A**	Conservative management of dens evaginatus and attached supernumerary tooth/odontome in mandibular premolar with dual radiolucencies. *Contemp Clin Dent* **6**(Suppl 1), S269-273	2015					X				
**Lin LM, Kim SG, Martin G, Kahler B.**	Continued root maturation despite persistent apical periodontitis of immature permanent teeth after failed regenerative endodontic therapy. *Aust Endod J* **44**(3),292-299	2018	X								
**Seirawan MY, Layous K, Seirawan MK, Doumani M**	Coronal discoloration related to bioceramic and mineral trioxide aggregate coronal barrier in non-vital mature teeth undergoing regenerative endodontic procedures. *World Journal of Dentistry* **11** (1), 52-60	2020							X		
**Thier M, Bußmeier U, Langenhan J, Kopp S**	Craniomandibular dysfunction in therapy with intraoral advancement splints. *Manuelle Medizin* **52**(6),521-526	2014							X		
**Pilbauerová N, Suchánek J**	Cryopreservation of Dental Stem Cells. *Acta Medica* **61**(1) 1-7	2018							X		
**Ma L, Makino Y, Yamaza H, Akiyama K, Hoshino Y, Song G *et al.***	Cryopreserved dental pulp tissues of exfoliated deciduous teeth is a feasible stem cell resource for regenerative medicine. *PLoS One* **7**(12), e51777	2012		X							
**Matoug-Elwerfelli M**	Decellularisation of the dental pulp for use as a scaffold in regenerative endodontics.	2017							X		
**Chrepa V, Henry MA, Daniel BJ, Diogenes A**	Delivery of apical mesenchymal stem cells into root canals of mature teeth. *J Dent Res* **94**(12), 1653-1659.	2015							X		
**Martínez-Sarrà E, Montori S, Gil-Recio C, Sampaolesi M, Atari M *et al.***	Dental pulp stem cells promote wound healing and muscle regeneration. *Stem Cell Genetics for Biomedical Research: Past, Present, and Future,* 221-240	2018							X		
**Botelho J, Cavacas MA, Machado V, Mendes JJ**	Dental stem cells: recent progresses in tissue engineering and regenerative medicine. *Annals of medicine*, **49**(8), 644–651	2017							X		
**Hirayama M, Oshima M, Tsuji T**	Development and prospects of organ replacement regenerative therapy. *Cornea* **32**, S13-S2	2013						X			
**Otsu K, Kishigami R, Oikawa-Sasaki A, Fukumoto S, Yamada A, Fujiwara N *et al.***	Differentiation of induced pluripotent stem cells into dental mesenchymal cells. *Stem Cells Dev* **21**(7), 1156-1164	2012			X						
**NCT03652129**	Digitized Assessment for the Survival of Mature Anterior Teeth With Periapical Lesion After One Step Regenerative Approach Using Different Asepsis Maneuvers	2018								X	
**Latham J, Fong H, Jewett A, Johnson JD, Paranjpe A.**	Disinfection Efficacy of Current Regenerative Endodontic Protocols in Simulated Necrotic Immature Permanent Teeth. *J Endod* **42**(8), 1218-1225	2016	X								
**Paino F, Ricci G, De Rosa A, Tirino V, Papaccio G**	Ecto-mesenchymal stem cells from dental pulp are committed to differentiate into active melanocytes. *European Cells and Materials* **20**, 295-305	2010			X						

Continue

Author	Reference	Year	Open Apex	Primary dentition	In vitro	Animal	Case Reports	Review	Of Topic	Cochrane Abstracts	Select
**NCT04313010**	Effectiveness of Regenerative Endodontics Therapy for Single-rooted Mature Permanent Tooth With Pulp Necrosis	2020								X	
**Hill, ST**	Effects of DynaMatrix on Angiogenic Cytokine and Matrix Metalloproteinase Expression from Human Endothelial Cells: An In-vitro Study.	2015			X						
**Bastidas C, Magda Z, Alvarez A, Diana P, Vélez L, Elenor M *et al.***	El rol de la pasta triple antibiótica en endodoncia regenerativa: una revisión bibliográfica/ The role of the triple antibiotic paste in regenerative endodontics: a review of the literature. *Rev. Asoc. Odontol. Argent* **105**(3), 123-132	2017						X			
**NCT03102879**	Encapsulated Mesenchymal Stem Cells for Dental Pulp Regeneration	2017							X		
**Jung, M**	Endodontics in the immature permanent dentition - Vital pulp therapy, apexification and regeneration [Article@Endodontie in der unreifen bleibenden Dentition - Maßnahmen zur Vitalerhaltung, Apexifikation und Regeneration der Pulpa]. *Oralprophylaxe und Kinderzahnheilkunde* **38**(1), 29-45	2016	X								
**Bucchi C, Gimeno-Sandig A, Manzanares-Céspedes C**	Enlargement of the apical foramen of mature teeth by instrumentation and apicoectomy. A study of effectiveness and the formation of dentinal cracks. *Acta Odontologica Scandinavica* **75**(7), 488-495	2017							X		
**Tomokazu H, Naoyuki C, Takeyoshi A, Yoshitaka Y, Akira I, Mitsuro T**	Establishment of clonal periodontal ligament cell line derived from deciduous tooth immortalized by human telomerase reverse transcriptase (hTERT) gene transfer. *Interface Oral Health Science*, 114-116	2011			X						
**Boisvert, M**	Étude de l'effet d'une matrice à base de collagène sur la différenciation des cellules souches de la pulpe dentaire en odontoblastes. Université Laval, Thèses et mémoires.	2019							X		
**Linsuwanont P, Sinpitaksakul P, Lertsakchai T**	Evaluation of root maturation after revitalization in immature permanent teeth with nonvital pulps by cone beam computed tomography and conventional radiographs. *International Endodontic Journal* **50**(9), 836–846	2016	X								
**Gebhardt, M**	Evaluation of tissue engineering scaffolds for regenerative endodontic treatment	2008							X		
**Bucchi Morales MC**	Experimental Approaches for Pulp Tissue Regeneration. PhD Thesis.	2019			X						
**Yang JZ, Ouyang Y, Liao Q**	Experimental study on pulp revitalization of Beagle dog's immature permanent teeth after pulpectomy. *Chinese Journal of Stomatology* **46**(8),489-493	2011				X					
**Jayaraman P, Govindasamy V, Gnanasegaran N, Kunasekaran W, Vasanthan P, Musa S**	Expression patterns of immune genes in long-term cultured dental stem cells., *Clinical Oral Investigations* **20**(1), 109–116.	2015			X						

Continue

Author	Reference	Year	Open Apex	Primary dentition	In vitro	Animal	Case Reports	Review	Of Topic	Cochrane Abstracts	Select
**Hirayama M, Tsubota K, Tsuji T**	Functional lacrimal gland regeneration. *Organ Regeneration Based on Developmental Biology*, 135-151	2017							X		
**Oshima M, Mizuno M, Imamura A, Saito M, Tsuj, T.**	Functional tooth regeneration using a bioengineered tooth unit as a mature organ replacement regenerative therapy. *PLoS ONE*, **6**(7), e21531	2011				X					
**Mimeault M, Batra SK**	Great promise of tissue-resident adult stem/progenitor cells in transplantation and cancer therapies. Advances in Experimental Medicine and Biology **741**, 171-186	2012							X		
**Fouad AF, Verma P**	Healing after regenerative procedures with and without pulpal infection. *Journal of Endodontics* **40**(4 SUPPL.), S58-S64	2014						X			
**Shiehzadeh F, Shiehzadeh V, Aghmasheh F, Joulae M, Kosarieh E *et al.***	Healing of large periapical lesions following delivery of dental stem cells with an injectable scaffold: New method and three case reports. *Indian Journal of Dental Research* **25**(2), 248-253	2014					X				
**Digka A, Sakka D, Lyroudia K**	Histological assessment of human regenerative endodontic procedures (REP) of immature permanent teeth with necrotic pulp/apical periodontitis: A systematic review. *Australian endodontic journal* **46** (1), 140-153.	2020						X			
**Arslan H, Şahin Y, Topçuoğlu HS, Gündoğdu B**	Histologic Evaluation of Regenerated Tissues in the Pulp Spaces of Teeth with Mature Roots at the Time of the Regenerative Endodontic Procedures. *Journal of Endodontics* **45**(11), 1384-1389	2019					X				
**Breivik M**	Human odontoblast response to tooth replantation. *European Journal of Orthodontics* **3**(2), 95-108	1981							X		
**Czerniuk MR, Zaremba, M**	Immediate implant placement after extraction of central incisor in the maxilla after injury - Case report. *Dental and Medical Problems* **49**(4), 600-606	2012							X		
**Zhu X, Wang Y, Liu Y, Huang GT-J, Zhang C**	Immunohistochemical and histochemical analysis of newly formed tissues in root canal space transplanted with dental pulp stem cells plus platelet-rich plasma. *Journal of Endodontics* **40**(10), 1573-1578	2014			X						
**Tamaki Y, Nakahara T, Ishikawa H, Sato S**	In vitro analysis of mesenchymal stem cells derived from human teeth and bone marrow. *Odontology* **101**(2), 121–132.	2012			X						
**Oliveira AP**	Influência de medicações intracanal utilizadas em procedimentos endodônticos regenerativos na sobrevivência de células da papila apical in vitro / Influence of intracanal medications used in regenerative endodontic procedures on survival of apical papilla cells in vitro. Master's Dissertation.	2015			X						
**Duggal M, Tong HJ, Al-Ansary M, Twati W, Day PF, Nazzal H**	Interventions for the endodontic management of non-vital traumatised immature permanent anterior teeth in children and adolescents: a systematic review of the evidence and guidelines of the European Academy of Paediatric Dentistry. *European Archives of Paediatric Dentistry* **18**(3), 139–151	2017						X			
**Ahangari Z, Nasser M, Mahdian M,**	Interventions for the management of external root resorption. *Cochrane Database of Systematic Reviews*.	2015							X		

Continue

Author	Reference	Year	Open Apex	Primary dentition	In vitro	Animal	Case Reports	Review	Of Topic	Cochrane Abstracts	Select
**Fedorowicz Z, Marchesan MA**											
**Tsilingaridis G, Malmgren B, Andreasen JO, Malmgren O**	Intrusive luxation of 60 permanent incisors: a retrospective study of treatment and outcome. *Dental Traumatology* **28**(6), 416-422	2012							X		
**Raymond R**	Investigation of new regenerative endodontic therapies utilizing in vitro and non-human primate models.	2011				X					
**Cielecki M**	Investigation of the effects of oral pathogens, antibiotics, and disinfectants on regenerative endodontic treatment.	2011							X		
**Fahmy SH, Hassanien EES, Nagy MM, El Batouty KM, Mekhemar M, Fawzy El Sayed K,Dörfer C et al.**	Investigation of the regenerative potential of necrotic mature teeth following different revascularisation protocols. *Australian Endodontic Journal* **43**(2), 75-84	2017				X					
**Chen Y-P, Jovani-Sancho M del M, Sheth CC**	Is revascularization of immature permanent teeth an effective and reproducible technique*? Dent Traumatol* **31**(6), 429-436	2015	X								
**Pelaez D, Huang C-YC, Cheung HS**	Isolation of pluripotent neural crest-derived stem cells from adult human tissues by connexin-43 enrichment. *Stem Cells and Development* **22**(21), 2906-2914	2013							X		
**Maria OM, Khosravi R, Mezey E, Tran SD**	Cells from bone marrow that evolve into oral tissues and their clinical applications. *Oral Diseases* **13**(1), 11-16	2007							X		
**Chan EKM, Desmeules M, Cielecki M, Dabbagh B, Ferraz dos Santos B**	Longitudinal Cohort Study of Regenerative Endodontic Treatment for Immature Necrotic Permanent Teeth. *Journal of Endodontics* **43**(3), 395-400	2017	X								
**Weigel C, Bragger U, Hammerle CHF, Mombelli A, Lang NP**	Maintenance of new attachment 1 and 4 years following guided tissue regeneration (GTR). *Journal of Clinical Periodontology* **22**(9), 661–669	1995							X		
**Verma PK, Srivastava R, Gupta KK, Srivastava A**	Management of combined endodontic - periodontal lesion: Case reports. *Medico-Legal Update* **12**(1), 107-109	2012					X				
**Saoud TMA, Huang GT-J, Gibbs JL, Sigurdsson A, Lin LM**	Management of immature necrotic permanent teeth with regenerative endodontic procedures - a review of literature. *Journal of Endodontics* **41**(10), 1743-1748	2015						X			
**Moodley DS, Peck C, Moodley T, Patel N**	Management of necrotic pulp of immature permanent incisor tooth: A regenerative endodontic treatment protocol: case report. *SADJ* **72** (3), 122 - 125	2017					X				
**Zhao J-H, Tsai C-H, Chang Y-C**	Management of radicular cysts using platelet-rich fibrin and bioactive glass: A report of two cases. *Journal of the Formosan Medical Association* **113**(7), 470-476	2014					X				

Continue

Author	Reference	Year	Open Apex	Primary dentition	In vitro	Animal	Case Reports	Review	Of Topic	Cochrane Abstracts	Select
**Saoud TMA, Huan,GT.-J, Gibbs JL,Sigurdsson A, Lin LM**	Management of teeth with persistent apical periodontitis after root canal treatment using regenerative. *Journal of Endodontics*, **41**(10), 1743-1748	2015					X				
**Honda MJ, Watanabe E, Mikami Y, Saito Y, Toriumi T, Shirakawa T *et al.***	Mesenchymal dental stem cells for tissue regeneration. The International *Journal of Oral & Maxillofacial Implant* **28**(6), e451–e460	2013							X		
**Bucchi C, de-Anta JM, Manzanares-Céspedes MC.**	Micro-Computed Tomographic Analysis of Apical Foramen Enlargement of Mature Teeth: A Cadaveric Study - Análisis Mediante Micro-CT del Ensanchamiento del Foramen Apical de Dientes Maduros: un Estudio Cadavérico. *Int. j. odontostomatol.***14**(2), 177-182	2020							X		
**Vidovic Zdrilic I, de Azevedo Queiroz IO, Matthews BG, Gomes-Filho JE, Mina M, Kalajzic I**	Mineral trioxide aggregate improves healing response of periodontal tissue to injury in mice. *Journal of Periodontal Research* **52**(6), 1058–1067	2017				X					
**Park J, Lee Y, Shin J, Lee HJ, Son YB, Park BW *et al.***	Mitochondrial genome mutations in mesenchymal stem cells derived from human dental induced pluripotent stem cells. *BMB Rep* **52**(12), 689-694	2019							X		
**Brignardello-Petersen R**	Outcomes of platelet concentrates for treating immature permanent teeth are acceptable but do not seem to be better than those of other techniques. *Journal of the American Dental Association* 150(5), E56	2019	X								
**Meza G, Urrejola D, Saint Jean N, Inostroza C, López V, Khoury M**	Personalized Cell Therapy for Pulpitis Using Autologous Dental Pulp Stem Cells and Leukocyte Platelet-rich Fibrin: A Case Report. *Journal of Endodontics* **45**(2), 144-149.	2019					X				
**Mishra N,Narang I, Mittal N**	Platelet-rich fibrin-mediated revitalization of immature necrotic tooth. *Contemp Clin Dent* **4**(3), 412-415	2013	X								
**Haque N, Abu Kasim NH**	Pooled Human Serum Increases Regenerative Potential of In Vitro Expanded Stem Cells from Human Extracted Deciduous Teeth. *Stem Cells: Biology and Engineering* 29-44	2017			X						
**Yam GHF, Teo EPW, Setiawan M, Lovatt MJ, Yusoff NZBM, Fuest M**	Postnatal periodontal ligament as a novel adult stem cell source for regenerative corneal cell therapy. *Journal of Cellular and Molecular Medicine* **22**(6), 3119-3132	2018							X		
**Chandrahasa S, Murray PE, Namerow KN**	Proliferation of mature ex vivo human dental pulp using tissue engineering scaffolds. *Journal of Endodontics* **37**(9), 1236-1239	2011			X						
**Yamada Y, Ito K, Nakamura S, Ueda M, Nagasaka T**	Promising cell-based therapy for bone regeneration using stem cells from deciduous teeth, dental pulp, and bone marrow. *Cell Transplantation* **20**(7), 1003-1013	2011		X							

Continue

Author	Reference	Year	Open Apex	Primary dentition	In vitro	Animal	Case Reports	Review	Of Topic	Cochrane Abstracts	Select
**St Paul A, Phillips C, Lee JY. Khan AA**	Provider Perceptions of Treatment Options for Immature Permanent Teeth. *Journal of Endodontics* **43**(6), 910-915	2017	X								
**Harini PM, Tambakad PB, Naidu J**	Pulp and Periodontal Regeneration of an Avulsed Permanent Mature Incisor Using Platelet-rich Plasma after Delayed Replantation: A 12-month Clinical Case Study. *Journal of Endodontics* **42**(1), 66-71	2016					X				
**Hargreaves KM, Diogenes A, Teixeira FB**	Pulp Injury and Changing Trends in Treatment. *Stem Cell Biology and Tissue Engineering in Dental Sciences,* 397-404	2015							X		
**Orti V, Collart-Dutilleul PY, Piglionico S, Pall O, Cuisinier F, Panayotov I**	Pulp regeneration concepts for nonvital teeth: From tissue engineering to clinical approaches. *Tissue Engineering - Part B: Reviews* **24**(6), 419-442	2018						X			
**Yang J, Yuan G, Chen Z**	Pulp Regeneration: Current Approaches and Future Challenges. *Frontiers in Physiology* **7**	2016						X			
**Kratunova E, Silva D**	Pulp therapy for primary and immature permanent teeth: an overview. *General Dentistry* **66**(6), 30-38	2018						X			
**El-Kateb NM, El-Backly RN, Amin WM, Abdalla AM**	Quantitative Assessment of Intracanal Regenerated Tissues after Regenerative Endodontic Procedures in Mature Teeth Using Magnetic Resonance Imaging: A Randomized Controlled Clinical Trial. *Journal of Endodontics* **46**(5), 563-574	2020									X
**Saoud TMA, Ricucci D, Lin LM, Gaengler P**	Regeneration and Repair in Endodontics-A Special Issue of the Regenerative Endodontics-A New Era in Clinical Endodontics. *Dent J (Basel)* **4**(1), 3.	2016						X			
**Roshene R**	Regeneration of dental pulp- A review. *Journal of Pharmaceutical Sciences and Research* **7(**10), 858-860	2015						X			
**Hargreaves KM, Giesler T, Henry M, Wang Y**	Regeneration Potential of the Young Permanent Tooth: What Does the Future Hold? *Pediatric Dentistry* **30**(3), 253-260	2008	X								
**Saoud TMA, Mistry S, Kahler B, Sigurdsson A, Lin LM**	Regenerative Endodontic Procedures for Traumatized Teeth after Horizontal Root Fracture, Avulsion, and Perforating Root Resorption. *Journal of Endodontics* **42**(10), 1476-1482	2016							X		
**Arslan H, Ahmed HMA, Şahin Y, Doğanay Yıldız E, Gündoğdu EC, Güven Y, Khalilov R**	Regenerative Endodontic Procedures in Necrotic Mature Teeth with Periapical Radiolucencies: A Preliminary Randomized Clinical Study. *Journal of Endodontics* **45**(7), 863-872	2019									X
**Tong HJ, Rajan S, Bhujel N, Kang J, Duggal M, Nazzal H**	Regenerative Endodontic Therapy in the Management of Nonvital Immature Permanent Teeth: A Systematic Review—Outcome Evaluation and Meta-analysis. *Journal of Endodontics* **43**(9), 1453-1464	2017						X			
**Chueh L-H, Ho Y-C, Kuo T-C, Lai W-H, Chen Y-HM, Chiang C-P**	Regenerative Endodontic Treatment for Necrotic Immature Permanent Teeth . *J Endod* **35**(2), 160-4	2009	X								

Continue

Author	Reference	Year	Open Apex	Primary dentition	In vitro	Animal	Case Reports	Review	Of Topic	Cochrane Abstracts	Select
**Xu Q, Li,Z**	Regenerative Endodontic Treatment of a Maxillary Mature Premolar. *Case Reports in Dentistry* (12), 1-5	2018					X				
**NCT04018456**	Regenerative Endodontic Treatment of Mature Necrotic Teeth With Apical Periodontitis	2019								X	
**Paryani K, Kim SG**	Regenerative endodontic treatment of permanent teeth after completion of root development: A report of 2 cases. *Journal of Endodontics* **39**(7), 929-934	2013					X				
**He L, Kim SG, Gong Q, Zhong J, Wang S, Zhou X *et al.***	Regenerative Endodontics for Adult Patients. *Journal of Endodontics* **43**(9), S57-S64	2017						X			
**Rosa V, Botero TM, Nör JE**	Regenerative endodontics in light of the stem cell paradigm. *International Dental Journal* 61, 23–28	2011						X			
**Bansal R, Bansal R**	Regenerative endodontics: A state of the art. *Indian Journal of Dental Research* **22** (1), 122-131	2011						X			
**Chatha, NR**	Regenerative endodontics: chart review of treated cases. Thesis of Master of Science in Dentistry	2017						X			
**Vieira WA, McCusker CD**	Regenerative models for the integration and regeneration of head skeletal tissues. *Int. J. Mol. Sci* **19**(12), 3752	2018							X		
**Trope M**	Regenerative Potential of Dental Pulp. *Pediatric Dentistry* **30** (3), 206-210	2008							X		
**Howard C**	Regulation of dental pulp stem cell migration and regenerative endodontics.Thesis.	2010							X		
**Pohl Y, Filippi A, Kirschner H**	Results after replantation of avulsed permanent teeth. II. Periodontal healing and the role of physiologic storage and antiresorptive-regenerative therapy. *Dental Traumatology* **21**(2), 93-101	2005							X		
**Pohl Y, Wahl G, Filippi A, Kirschner H**	Results after replantation of avulsed permanent teeth. III. Tooth loss and survival analysis. *Dental Traumatology* **21**(2), 102-110	2005							X		
**Correia TRO**	Revascularização pulpar. Master thesis.	2018						X			
**Moreu L**	Revascularización pulpar: presentación de un caso clínico/ Pulp revascularization: report of a clinical case. *Rev. Fac. de Odon. UBA*	2018					X				
**Sridharan S, Neelakantan I,Neelakantan P**	Revascularization in endodontics. *International Journal of Clinical Dentistry* **7**(2), 139-145.	2014						X			
**Neelamurthy, P.S., Kumar RA, Balakrishnan V, Narayan GS, Karthikeyan I**	Revascularization in Immature and Mature Teeth with Necrotic Pulp: A Clinical Study. *Journal of Contemporary Dental Practice* **19**(11), 1394-1400	2018	X								
**Cehreli ZC, Sara S, Aksoy B**	Revascularization of immature permanent incisors after severe extrusive luxation injury. *Journal of the Canadian Dental Association* **78**(1), c4	2012	X								

Continue

Author	Reference	Year	Open Apex	Primary dentition	In vitro	Animal	Case Reports	Review	Of Topic	Cochrane Abstracts	Select
**Moreno-Hidalgo MC, Caleza-Jimenez C, Mendoza-Mendoza A, Iglesias-Linares A**	Revascularization of immature permanent teeth with apical periodontitis. *Int Endod J* **47**(4), 321-331	2014	X								
**Wigler R, Kaufman AY, Lin S, Hazan-Molina H, Torneck CD**	Revascularization: A treatment for permanent teeth with necrotic pulp and incomplete root development. *Journal of Endodontics* **39**(3), 319-326	2013	X								
**Nagas EM, Uyanik O, Cehreli ZC**	Revitalization of necrotic mature permanent incisors with apical periodontitis: a **case** report. *Restor Dent Endod* **43**(3), e31	2018					X				
**CTRI/2012/11/003102**	Root canal treatment without obturation with regenerative technique	2012								X	
**Shah N, Logani A**	SealBio: A novel, non-obturation endodontic treatment based on concept of regeneration. *J Conserv Dent* **15**(4), 328-332	2012									X
**Yoshimi R, Yamada Y, Ito K, Baba S, Ueda M**	Self-assembling peptide nanofiber scaffolds, platelet-rich plasma, and mesenchymal stem cells for injectable bone regeneration with tissue engineering. *Journal of Craniofacial Surgery* **20**(5), 1523-1530	2009							X		
**Alkilzy M, Tarabaih A, Santamaria RM, Splieth CH**	Self-assembling Peptide P(11)-4 and Fluoride for Regenerating Enamel. *Journal of Dental Research* **97**(2), 148-154	2018							X		
**Altaii M, Broberg M, Cathro P, Richards L**	Standardisation of sheep model for endodontic regeneration/revitalisation research. *Archives of Oral Biology* **65**, 87-94	2016				X					
**Yeasmin S, Ceccarelli J, Vigen M, Tarle SA, Kaigler D**	Stem cells derived from tooth periodontal ligament enhance functional angiogenesis by endothelial cells. *Tissue Engineering - Part A* **20**(7-8), 1188-1196	2014			X						
**Martinez Saez D, Sasaki RT, Neves AC, · da Silva MCP**	Stem Cells from Human Exfoliated Deciduous Teeth: A Growing Literature. *Cells Tissues Organs* **16(**202),269–280	2015		X							
**Khazaei M, Bozorgi A, Khazaei S, Khademi A**	Stem cells in dentistry, sources, and applications. *Dental Hypotheses* **7**(2), 42-52.	2016							X		
**NCT03717337**	Success Rate of Single Versus Two Visit Regenerative Treatment Protocol in Non Vital Mature Anterior Teeth	2018								X	
**Chrepa V, Pitcher B, Henry MA, Diogenes A**	Survival of the Apical Papilla and Its Resident Stem Cells in a Case of Advanced Pulpal Necrosis and Apical Periodontitis. *Journal of Endodontics* **43**(4), 561-567	2017			X						
**Chrepa V, Pitcher B, Henry MA, Diogenes A**	Transforming Growth Factor Beta 1 Distribution and Content in the Root Dentin of Young Mature and Immature Human Premolars	2017			X						
**Chien Y, Chang YL, Li HY, Lee SD, Huang P**	Synergistic effects of carboxymethyl-hexanoyl chitosan, cationic polyurethane-short branch PEI in miR122 gene delivery: Accelerated differentiation of iPSCs into mature	2015							X		

Continue

Author	Reference	Year	Open Apex	Primary dentition	In vitro	Animal	Case Reports	Review	Of Topic	Cochrane Abstracts	Select
	hepatocyte-like cells and improved stem cell therapy in a hepatic failure model. *Acta Biomaterialia* **13**, 228-244										
**D'Souza RN, Regan KR, Fisher JP, Mikos AG**	The bioengineering of dental tissues .	2012							X		
**Laureys WGM, Cuvelier CA, Dermaut LR, De Pauw GAM**	The critical apical diameter to obtain regeneration of the pulp tissue after tooth transplantation, replantation, or regenerative endodontic treatment. *Journal of Endodontics* **39**(6), 759-763	2013			X						
**Tamaoki N, Takahashi K, Aoki H, Yamanaka S, Tezuka K-I**	The homeobox gene DLX4 promotes generation of human induced pluripotent stem cells. *Scientific Reports* **4**,7283	2014			X						
**Biggerstaff R**	The residual antibacterial effects of radiopaque double antibiotic paste after various treatment times. Master Thesis	2018							X		
**Kim RH, Mehrazarin S, Kang MK**	Therapeutic Potential of Mesenchymal Stem Cells for Oral and Systemic Diseases. *Dental Clinics of North America* **56**(3), 651-675	2012							X		
**Schreurs M., Suttorp CM, Mutsaers HA , Kuijpers-Jagtman AM, den Hoff JW, Ongkosuwito EM *et al.***	Tissue engineering strategies combining molecular targets against inflammation and fibrosis, and umbilical cord blood stem cells to improve hampered muscle and skin regeneration following cleft repair. *Medicinal Research Reviews* **40**(1), 9-26.	2020							X		
**Langova P, Stembirek J, Matalova E, Buchtova M**	Tooth autotransplantations - lessons from animal models: a review. *Veterinarni medicina* **60**(6),293-300	2015						X			
**Yelick PC, Sharp PT**	Tooth Bioengineering and Regenerative Dentistry. *Journal of dental research* **98** (11), 1173-1182	2019							X		
**Brizuela C, Urrejola D, Meza G, Angelopoulos I, Khoury M**	Translational pathway of scalable, allogenic encapsulated mesenchymal stem cells for dental pulp regeneration. Ranokure a controlled phase i/ii clinical trial designed to evaluate the survival of mature permanent teeth with apical lesion following a regenerative endodontics procedure. Cytotherapy **20**(5), S93–S94	2018							X		
**Saoud TMA, Sigurdsson A, Rosenberg PA, Lin LM, Ricucci D**	Treatment of a large cyst like inflammatory periapical lesion associated with mature necrotic teeth using regenerative endodontic therapy. *Journal of Endodontics* **40**(12), 2081-2086	2014					X				
**Saoud TM, Martin G, Chen YHM, Chen KL, Chen CA, Songtrakul K**	Treatment of Mature Permanent Teeth with Necrotic Pulps and Apical Periodontitis Using Regenerative Endodontic Procedures: A Case Series. *Journal of Endodontics* **42**(1), 57-65	2016					X				
**CTRI/2019/04/018503**	Treatment of permanent teeth with diseased pulp using natural regeneration technique to regain pulp sensation and natural defense mechanism for pulp	2019								X	

Continue

Author	Reference	Year	Open Apex	Primary dentition	In vitro	Animal	Case Reports	Review	Of Topic	Cochrane Abstracts	Select
**Oshima M, Tsuji T**	Whole tooth regeneration as a future dental treatment. *Advances in Experimental Medicine and Biology* **881**, 255-269	2015						X			
**ElSheshtawy AS, Nazzal H, El Shahawy OI, El Baz AA, Ismail SM, Kang J**	The effect of platelet-rich plasma as a scaffold in regeneration/revitalization endodontics of immature permanent teeth assessed using 2-dimensional radiographs and cone beam computed tomography: a randomized controlled trial. *International endodontic journal* **53**(7), 905-921	2020	X								

Continue
