# Supplementary material for: Prognosis of Regenerative Endodontic Procedures in Mature Teeth: A Systematic Review and Meta-Analysis of Clinical and Radiographic Parameters
Source: Materials (Basel). 2021 Aug 6;14(16):4418. doi: 10.3390/ma14164418 (PMC8398537; doi:10.3390/ma14164418)
Supplement: Supplementary file 1 [file materials-14-04418-s001.zip › REP - Table S3.pdf]

Prognosis of regenerative endodontic procedures in mature teeth: a systematic review and meta-analysis of clinical and ra-diographic parameters

Table S3. Quality Assessment Tool for Before-After (Pre-Post) Studies

	Brizuela et al. 2020 [37]	El-Kateb et al. 2020 [39]	Arslan et al. 2019 [35]	Jha et al. 2019 [36]	Nageh *et al.* 2018 [34]	Shah 2016 [33]	Shah & Logani 2012 [31]
1. Was the study question or objective clearly stated?	Y	Y	Y	Y	Y	Y	N
2. Were eligibility/selection criteria for the study population prespecified and clearly described?	Y	Y	Y	Y	Y	Y	NR
3. Were the participants in the study representative of those who would be eligible for the test/service/intervention in the general or clinical population of interest?	Y	Y	Y	Y	Y	Y	Y
4. Were all eligible participants that met the prespecified entry criteria enrolled?	NR	Y	Y	NR	NR	NR	NR
5. Was the sample size sufficiently large to provide confidence in the findings?	N	Y	N	N	N	N	N
6. Was the test/service/intervention clearly described and delivered consistently across the study population?	Y	Y	Y	Y	Y	Y	Y
7. Were the outcome measures prespecified, clearly defined, valid, reliable, and assessed consistently across all study participants?	Y	Y	Y	Y	Y	N	N
8. Were the people assessing the outcomes blinded to the participants' exposures/interventions?	Y	Y	Y	Y	N	N	N
9. Was the loss to follow-up after baseline 20% or less? Were those lost to follow-up accounted for in the analysis?	Y	Y	Y	Y	Y	Y	N
10. Did the statistical methods examine changes in outcome measures from before to after the intervention? Were statistical tests done that provided p values for the pre-to-post changes?	Y	Y	NR	Y	Y	N	N
11. Were outcome measures of interest taken multiple times before the intervention and multiple times after the intervention (i.e., did they use an interrupted time-series design)?	N	N	N	N	N	N	N
12. If the intervention was conducted at a group level (e.g., a whole hospital, a community, etc.) did the statistical analysis take into account the use of individual-level data to determine effects at the group level?	NA	NA	NA	NA	NA	NA	NA
QUALITY RATING	GOOD	GOOD	GOOD	GOOD	FAIR	FAIR	POOR

*CD, cannot determine; NA, not applicable; NR, not reported; Y, Yes; N, No.
